# Navigating airports: a scoping review of physical access challenges and experiences for persons with disabilities

**DOI:** 10.3389/fresc.2026.1680067

**Published:** 2026-07-22

**Authors:** Ahmed Hadj Hassen, Ernesto Morales, David Gotti, Francois Routhier

**Affiliations:** 1 Department of Rehabilitation, Faculty of Medicine, Université Laval, Quebec City, QC, Canada; 2 Rehabilitation Sciences School of Rehabilitation - Faculty of Medicine, Université Laval, Quebec City, QC, Canada

**Keywords:** air transportation, airport accessibility, inclusive design, persons with disabilities (PWDs), physical environment, social participation

## Abstract

Airports present a uniquely complex environment for travelers with disabilities, where the promise of global mobility often collides with persistent physical, sensory, and procedural barriers. This scoping review synthesizes peer-reviewed and gray literature to examine the physical environment of airports and how it shapes access for persons with disabilities, including the interaction between spatial features and service-related components in real-world travel situations. Guided by Fougeyrollas’s five-dimensional framework of access availability, accessibility, acceptability, affordability, and usability the review examines how each dimension shapes real-world travel experiences from curb to gate. The results suggest that, in the reviewed studies, accessibility often appears fragmented across the airport journey, with infrastructures that may be present but not always usable, available, or meaningfully integrated into the travel experience. Access often relies on assistance-based models, and important gaps remain in addressing diverse user needs, particularly for individuals with invisible disabilities. At the same time, the literature highlights promising practices and ongoing improvements, including digital wayfinding tools, dedicated assistance services, and growing attention to inclusive design principles. Overall, this review emphasizes that accessibility cannot be reduced to regulatory compliance or the mere presence of infrastructure. Rather, it must be understood as a dynamic and continuous process shaped by the interaction between environments and diverse human needs throughout the airport journey.

## Introduction

In 2022, approximately 16% of the world’s population over 1.3 billion people lived with a disability (1). In Canada, nearly 27% of adults reported one or more disabilities that significantly shape their daily experiences (2). Yet behind these statistics are individuals whose opportunities to fully engage with their communities depend, to a certain extent, on the environments they navigate. Indeed, access extends far beyond mere entry into physical spaces; at its core, it represents the fundamental freedom of each person particularly those facing social, economic, geographic, or technological exclusion to participate meaningfully and without constraint in everyday life (3).

Recognized internationally by legal instruments, such as the United Nations Convention on the Rights of Persons with Disabilities (CRPD), access calls for environments that move beyond minimal compliance toward genuine inclusivity (4). People with disabilities do not constitute a homogeneous group. Their experiences of the built environment vary depending on their functional, sensory, cognitive, or invisible characteristics (3, 4). In airport environments, these differences translate into distinct and context-specific access needs. While some individuals may encounter challenges related to mobility and physical navigation, others may be more affected by difficulties in orientation, information processing, or sensory overload, particularly in large-scale, complex, and highly dynamic settings such as airports (4, 5). These variations influence how travelers interact with spatial layouts, circulation paths, signage systems, and service interfaces throughout the airport journey.

Airport accessibility has traditionally been examined from the perspective of physical mobility impairments, with most studies focusing on barriers affecting wheelchair users and other travelers with visible disabilities (6, 7). Yet airport travel can also present challenges for people living with cognitive, sensory, psychosocial, and neurodevelopmental conditions, particularly in relation to communication, orientation, information processing, and sensory stimulation (8). Despite growing attention to these issues, invisible disabilities remain less represented in both research and airport accessibility initiatives. As a result, the needs of these travelers are not always fully considered in the design of airport environments and services.

In this context, Patrick Fougeyrollas conceptualizes access as the outcome of continuous interactions between individuals and their environments. This perspective was developed to move beyond a purely technical or compliance-based understanding of accessibility and to provide a more comprehensive framework for capturing how environmental conditions interact with diverse human needs in real-life contexts, including complex public infrastructures such as airports. It is structured around five interdependent dimensions: (1) availability, (2) accessibility, (3) acceptability, (4) affordability, and (5) usability (5). Availability refers to whether the necessary infrastructures and services exist and remain operational when and where they are needed. Accessibility examines the spatial configuration, circulation paths, and interface design to ensure they accommodate diverse mobility, sensory, and cognitive needs (5). Acceptability considers the social-psychological climate, staff attitudes, cultural fit, and perceived safety that shape whether environments feel dignified and welcoming (5). Affordability encompasses both direct and indirect costs, including time and effort required to access services. Finally, usability addresses the intuitiveness and reliability of facilities and services, determining whether individuals can use them independently and confidently (5). By positioning accessibility as one dimension within a broader process of access, Fougeyrollas highlights the importance of considering how environmental conditions shape opportunities for participation and autonomy. This perspective is consistent with broader discussions in disability, transport, and tourism research that view mobility not only as movement through space, but also as a condition for social inclusion, participation, and the exercise of rights (3, 5).

Despite the growing recognition of accessibility as a multidimensional concept, its translation into real-world environments remains uneven, particularly in complex infrastructures such as airports. Airports, as major hubs of international mobility, are often described as stressful environments. Their large scale, labyrinthine terminals, long walking distances, and sometimes confusing signage can significantly complicate the travel experience (9, 10). In the reviewed studies, physical infrastructures are not always fully adapted to diverse user needs. Staircases without clearly accessible alternatives, heavy manual doors, poorly located elevators, or the absence of appropriate arrangements in waiting areas and service zones may render key spaces difficult or even impossible to access (11). Features such as armless seating or the absence of sensory-calming environments may further limit comfort, independence, and usability, particularly for some individuals with sensory or cognitive sensitivities (12). In addition, unintuitive signage and noisy environments are often reported as significant barriers for individuals with visual or cognitive impairments, restricting their ability to orient themselves and navigate airport spaces autonomously (12).

While most airports have developed initiatives to improve access, these efforts often remain fragmented and unevenly implemented. Mobility assistance services, for example, are widely available but typically require advance booking, which may limit spontaneous use and flexibility (13). More broadly, many accessibility measures continue to rely on compensatory approaches, such as staff assistance or wheelchair provision, rather than on inclusive and integrated design strategies (14). This reliance on individualized support may inadvertently reinforce marginalization by creating separate pathways for passengers with disabilities (5). In addition, the diversity of disability profiles is not always fully considered in the design of airport infrastructures, which can reduce the effectiveness of existing adaptations and contribute to situations of discomfort or exclusion (3).

While previous reviews have explored accessibility in airport environments, they have often focused on specific aspects such as service provision or user experience in isolation. In contrast, this review adopts a multidimensional approach grounded in Fougeyrollas’ framework, allowing for a more integrated analysis of how physical environments and related elements shape access across the entire airport journey.

In this context, this scoping review aims to examine the physical environment of airport settings and how it shapes access for persons with disabilities. While the primary focus is placed on the built environment such as spatial configurations, circulation systems, and physical interfaces the analysis also considers service-related components when they directly influence how this environment is experienced in practice. This approach reflects the understanding that access emerges from the interaction between spatial characteristics and operational elements that shape real-world navigation. More specifically, this review seeks to answer the following research question: how does the physical environment of airports, in interaction with service-related components, shape access and the travel experience for persons with disabilities across the different stages of the airport journey?

## Methodology

This study employs a scoping review methodology to comprehensively examine practices and measures aimed at improving access to airport environments for persons with disabilities, with a primary focus on the physical environment. While the emphasis is placed on spatial and infrastructural features, service-related components were explicitly considered when they directly influenced how the physical environment is accessed and experienced in practice. This approach reflects the understanding that access emerges from the interaction between physical configurations and operational elements in real-world airport contexts. Unlike systematic reviews, which synthesize precise findings in response to narrowly defined questions, scoping reviews enable the exploration of research diversity, identification of gaps, and mapping of key concepts within an evolving field (15). Similar approaches have been adopted in previous reviews examining accessibility and travel experiences for people with disabilities, particularly in airport and transportation contexts (12). This approach is particularly appropriate for the present study, as it provides a broad and structured overview of existing practices as well as persistent challenges related to airport accessibility. Our methodology follows the JBI Manual for Evidence Synthesis (15), which offers a structured framework for conducting scoping reviews. Both peer-reviewed and gray literature were included to ensure a comprehensive and balanced analysis of available evidence.

### Methodological framework

#### Scientific literature

A literature search strategy was developed in collaboration with a specialized librarian from Université Laval (Québec, Canada) to optimize the relevance and comprehensiveness of the results. This approach relied on carefully selected search terms and Boolean operators (AND, OR) to maximize both sensitivity and specificity (16). The selected terms included “accessibility,” “passengers with disabilities,” “airports,” “physical environment,” “barriers,” “facilitators,” “inclusive design,” and “universal design.” Truncation symbols (e.g., *) were used where appropriate to capture variations of key terms across databases.

The search was conducted across databases recognized for their coverage of accessibility, architecture, and rehabilitation, including MEDLINE (via PubMed), Scopus, Avery’s Index of Architectural Periodicals, Web of Science, and CINAHL. The search was limited to studies published from the year 2000 onward, as this period reflects the growing integration of accessibility considerations and inclusive design principles in transportation and built environment research. The final search was conducted in 2024. References were managed using Zotero, which facilitated duplicate removal and the application of predefined relevance filters. Two reviewers (A.H.H. and D.G.) independently screened the titles, abstracts, and full texts to ensure objectivity and reproducibility (17).

#### Selection of scientific articles

The article selection process was conducted in three stages, in accordance with established methodological recommendations for scoping reviews (15, 17). First, titles and abstracts were screened to exclude clearly irrelevant studies. Second, the remaining articles were assessed in full text based on predefined eligibility criteria. Finally, a consensus process between reviewers led to the final selection of included studies.

The Covidence platform (https://www.covidence.org) was used to facilitate the management and tracking of the selection process, including duplicate removal and collaborative screening. Two independent reviewers (A.H.H. and D.G.) evaluated each article, thereby reducing the risk of selection bias (18). Disagreements were resolved through discussion, and when necessary, a third reviewer (E.M.) was consulted to reach consensus. A PRISMA extension for Scoping Reviews (PRISMA-ScR) flow diagram was used to document the selection process, including the number of records identified, screened, and excluded at each stage.

#### Inclusion and exclusion criteria

The inclusion and exclusion criteria applied in this review are presented in Table 1.

**Table 1:** Inclusion/exclusion criteria.

Inclusion criteria	Exclusion criteria
Articles published from the year 2000 onward.	Studies focused on market analyses or air traffic.
Studies focusing on individuals living with disabilities.	Works exploring profitability, passenger volume, or topics related to aerospace.
Research centered on specific measures implemented in airports.	Publications of an advertising nature, protocols, literature reviews, books, or opinion pieces.
Analyses of the impacts related to the physical, architectural, and spatial accessibility of the airport environment.	Articles written in a language other than French or English.

### Gray literature

To complement peer-reviewed evidence and capture current practices related to airport accessibility, a gray literature search was conducted following PRISMA-ScR recommendations (17). Gray literature was intentionally limited to Canadian sources to ensure alignment with the broader focus of this research on the accessibility of Canadian airport environments. Given the variability in accessibility policies and practices across countries, this choice allowed for a more coherent and contextually grounded interpretation of findings. These documents were identified through targeted searches of institutional and governmental websites, including airport authorities, accessibility organizations, and relevant agencies, as well as through the consultation of reports cited in the scientific literature.

## Results

### Scientific articles included

**Figure 1:**
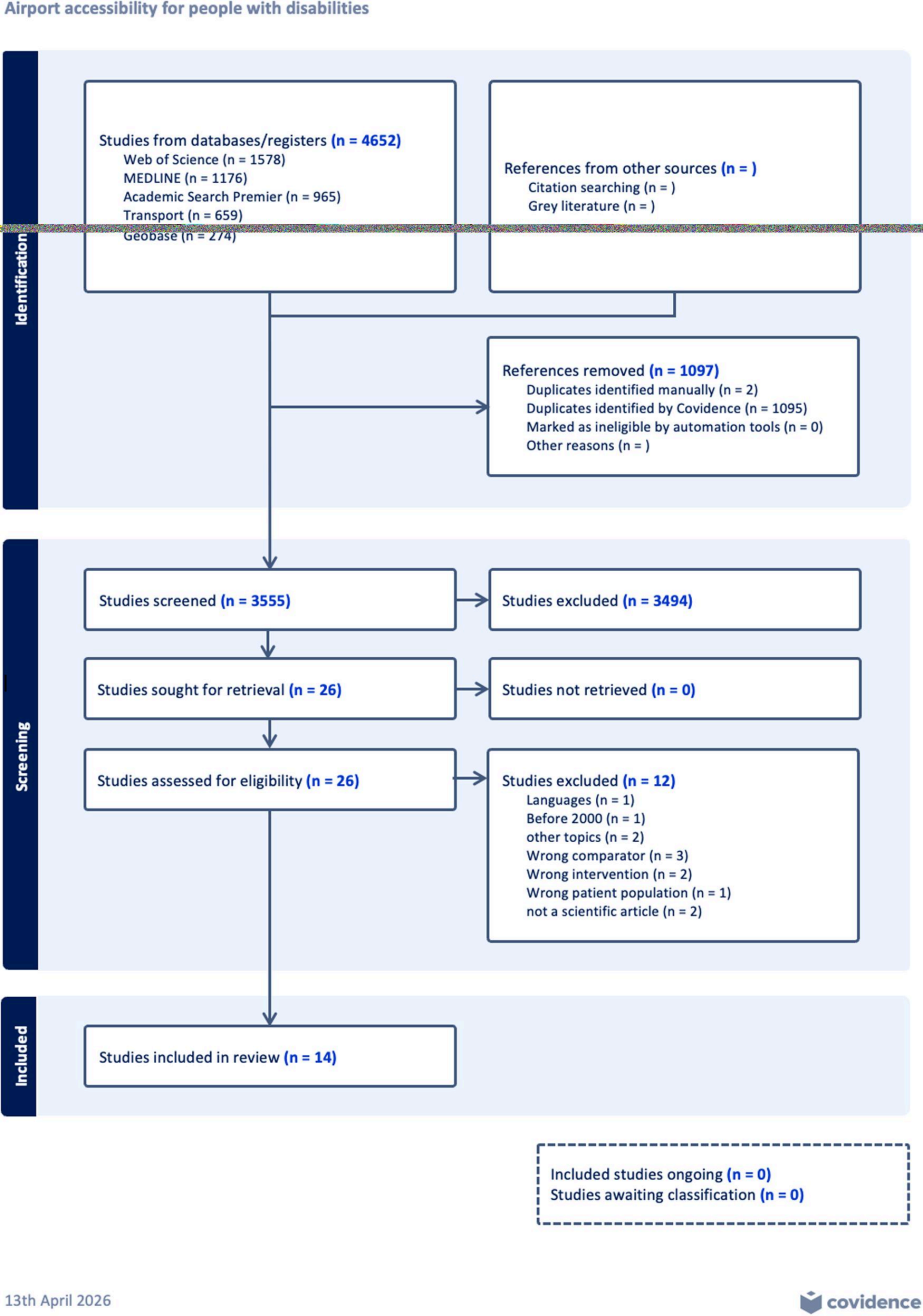
PRISMA-ScR diagram.

The article selection process, illustrated by a PRISMA extension for Scoping Reviews (PRISMA-ScR) diagram (Figure 1), initially identified 4,652 records. After duplicate removal, 3,555 unique records remained. Following title and abstract screening and full-text review, 14 articles were retained for analysis. Study designs included exploratory studies (*n* = 5), descriptive studies (*n* = 3), qualitative studies based on interviews or focus groups (*n* = 4), and quantitative studies (*n* = 2). The included studies covered a range of geographical contexts, including North America, Europe, Asia, and Australia, and examined different types of airport settings, from major international hubs to smaller regional facilities. They addressed a variety of disability profiles, including mobility, sensory, cognitive, and invisible disabilities, although some groups remained underrepresented, particularly individuals with neurocognitive conditions, psychosocial disabilities, and certain forms of sensory impairments such as hearing-related disabilities. Across the included studies, the focus was primarily on passenger navigation, access to services, and interactions with airport infrastructures at different stages of the travel journey. Taken together, these studies provide a broad overview of how accessibility is approached in diverse airport contexts, highlighting both common challenges and variations related to infrastructure, scale, and user needs.

### Gray literature included

In addition to the scientific articles, five gray literature documents were included in the analysis. These sources consisted of governmental reports, technical guidelines, and institutional publications focusing on airport accessibility in the Canadian context. The gray literature provided complementary insights into regulatory frameworks, implementation challenges, and emerging practices, particularly in relation to the implementation of accessibility measures in real-world airport environments.

### Data extraction

Data extraction was performed using a standardized form adapted from the JBI Manual for Evidence Synthesis (15) and pilot-tested on a subset of sources to ensure clarity and consistency. For each included document, information was collected on authorship, publication year, country or region, study design, population characteristics, documented physical accessibility features, and key findings. Particular attention was given to physical aspects of the airport environment, while also capturing service-related elements when they directly influenced how these environments were accessed and experienced in practice. Two reviewers independently extracted the data, and discrepancies were resolved through discussion until consensus was reached (18). Table A1 summarizes the main characteristics and key findings of the included studies. The full version of the table is available in Appendix 1.

### Data analysis

The collected data were analyzed using a mixed thematic approach combining both deductive and inductive coding (19). In the deductive phase, each data item was mapped to one of the five dimensions of access proposed by Fougeyrollas: availability, accessibility, acceptability, affordability, and usability (5). Particular attention was given to physical aspects of the airport environment, while also considering service-related elements when they directly influenced how access was experienced in practice. This approach reflects the understanding that access emerges from the interaction between spatial configurations and operational components in real-world airport contexts. In parallel, inductive coding was used to identify emergent sub-themes that were not fully captured by the initial framework. Coding and data organization were facilitated using NVivo software, ensuring traceability and transparency throughout the analytical process (20). Two reviewers (A.H.H. and D.G.) independently conducted the coding. Disagreements were resolved through discussion between the two reviewers, and when necessary, a third researcher (E.M.) was consulted to reach consensus (21). The resulting thematic structure, including categories, themes, and codes, is presented in Table 2.

**Table 2:** Categories, themes and codes used in the thematic content analysis.

Categories	Themes	Codes
Availability	Presence of infrastructures	Presence of ramps, elevators, accessible restrooms
	Availability of assistance services	Presence and availability of assistance services
	Orientation technologies	Integration of mobile beacons, audible beacons, interactive maps
Accessibility	Physical infrastructure	Presence of circulation pathways, access ramps, widened areas
	Staff Training	Lack of employee training on accessibility
	Signage and orientation	Clear signage, intuitive wayfinding
Acceptability	User Experience	Passenger satisfaction, travel-related stress
	Staff treatment	Staff training, attitudes, awareness
	Service Adaptation	Adaptation of services to different types of disabilities
Affordability	Cost of accessibility services	Free or paid accompaniment services
	Airport Investments	Investments in airport accessibility
	Access to Specialized Equipment	Financial accessibility of wheelchairs and specialized equipment
Usability	Ease of Use of Digital Tools	Ease of use of guidance applications
	Infrastructure Ergonomics	Ergonomic design of physical infrastructures

### Framing

Our analysis of airport infrastructure and services for passengers with disabilities unfolds across the five interlinked dimensions of access proposed by Fougeyrollas: availability, accessibility, acceptability, affordability, and usability, each offering a distinct lens through which to interpret how existing measures respond to travelers’ needs in the included studies (5). Although these dimensions are presented separately for analytical purposes, they remain closely interconnected in practice. A single feature may influence more than one dimension of access simultaneously. For example, an accessible facility may exist and be physically reachable, yet still be difficult to use or perceived negatively by users. The distinction between dimensions is therefore intended to support analysis rather than suggest clear-cut boundaries between lived experiences (5).

#### Availability

Assistance services are widely reported as being available in international airports, taking the form of wheelchair loans and accompanying personnel (19, 22, 23). However, despite their availability, these resources are sometimes limited in number or unevenly distributed across the airport space, particularly in peripheral areas or during certain times of day (24, 25). Several travelers have reported long wait times or the absence of staff upon arrival, which in some cases forced them to interrupt their journey or rely on informal support (26, 27).

As for physical infrastructure, many essential features are indeed present, including access ramps, elevators, accessible restrooms, and ergonomic seating (28, 29). Yet, these installations are often aging, sometimes out of order, or so poorly located that they become nearly unusable (24, 30). An elevator may be installed but out of service. A lowered counter may exist but be used for other purposes (30). Some resources are also subject to strict time-based conditions. Assistance services, for instance, may require a reservation up to 48hours in advance, limiting access for travelers who have not planned ahead or whose needs change rapidly (31, 32). Such constraints run counter to the unpredictable nature of certain journeys and may exclude users from the outset (33).

Digital technologies such as self-service kiosks, biometric gates, or navigation systems are becoming more common, but their presence remains uneven from one terminal to another (25, 34). In some cases, these features are available only in central areas, leaving transit zones, parking lots, or arrivals halls without equivalent solutions (34). Moreover, they are not always compatible with assistive tools such as screen readers or voice commands, significantly limiting their usability (35, 36). Key travel information has in some cases been reported as available in only one language or accessible primarily through digital formats, making its availability dependent on specific user abilities, whether linguistic, technological, or cognitive (30).

Certain targeted initiatives have been developed to address invisible disabilities, such as sunflower lanyards, which have been implemented in several airport contexts (33). While adopted in several airports, their availability remains inconsistent, and their effectiveness depends on staff awareness. Several travelers have reported wearing the lanyard without receiving any particular attention (37).

More broadly, issues related to the spatial distribution of accessibility resources persist across airport environments. Even in well-equipped terminals, significant gaps remain between central zones and more peripheral areas such as parking lots, secondary entrances, or drop-off areas (24). This spatial disparity creates gaps in the availability chain, rendering certain segments of the journey inaccessible, not necessarily due to a lack of intent, but due to the absence or uneven distribution of resources at a given location (26, 29, 38). At the same time, several studies highlight ongoing efforts to improve the distribution and reliability of accessibility features, reflecting a gradual shift toward more inclusive airport environments.

#### Accessibility

In the included studies, people with disabilities may encounter barriers as soon as they enter airport environments, particularly during the initial stages of the travel journey. When doors are too heavy, manual, or non-automated, physical access becomes dependent on outside assistance, especially for individuals with mobility impairments (31). Excessively steep ramps, the absence of handrails or non-slip surfaces, and stairs without an equivalent alternative are frequently cited as obstacles upon arrival (38, 39).

Inside the terminal, long distances between check-in, security, and boarding areas are particularly challenging when routes are fragmented or lack visual, tactile, or auditory cues (40). In many cases, adapted pathways are either poorly marked or fail to offer detours around obstacles, forcing some travelers to take longer or more circuitous routes (29). Entire segments of the journey, such as baggage claim areas, waiting zones, or security checkpoints, still present barriers: narrow queues, absence of accessible seating, counters that are too high or not adjustable (31, 39). Even when solutions such as accessible counters, quiet areas, or priority lanes do exist, their location, signage, and activation mechanisms are not always designed to support autonomous use (30, 39).

Signage, although essential for orientation, is too often designed without applying universal design principles. Pictograms may be unclear, fonts too small or low-contrast, and directional signs incomplete or poorly positioned (38). This lack of consistency in the visual environment often forces travelers to seek help, reducing their autonomy (39). The absence of sensory markers such as tactile ground indicators, audio beacons, or differentiated floor textures also impedes orientation for blind or low-vision individuals (29). The digital environment, increasingly relied upon for check-in and orientation processes, does not always offer equitable access. Interactive kiosks, often positioned for standing use, require fine motor control, rapid reading, or touch-based interaction that can exclude some users, particularly those with visual impairments (25). The absence of voice alternatives, sufficient visual contrast, or compatibility with assistive technologies makes the experience difficult or even impossible for many people (34, 35).

Where assistance is available, it often requires prior booking or an understanding of help-request mechanisms, which can itself be a barrier (28). Furthermore, assistance modalities, often centered on wheelchair users, may overlook other forms of less visible disabilities, such as sensory, cognitive, or psychosocial limitations (33, 37). At the same time, several studies highlight ongoing improvements in wayfinding systems and digital navigation tools, suggesting a gradual evolution toward more accessible airport environments, although these initiatives remain unevenly implemented.

#### Acceptability

In the included studies, the way airport spaces are designed directly shapes the experience of people with disabilities. Too often, facilities labeled as “accessible” impose routes that are humiliating, stigmatizing, or simply inadequate (21, 22). Entire sections are sometimes configured as parallel circuits, reserved for assistance and removed from the main passenger flow. Having to take a separate path, go through service doors, or wait in secondary areas can create a sense of exclusion (26, 33).

When an elevator is located at the end of a hallway, poorly lit or marked by a discreet pictogram, it sends a clear message: this amenity is not truly integrated; rather, it is tolerated (26, 30, 31). Many travelers report that so-called “accessible” restrooms are off to the side, locked, or cluttered, requiring assistance from a staff member or long waits for access to a basic necessity (38). Some areas are designed without consideration for users’ pacing or sensory needs. Overcrowded, noisy waiting areas without adapted seating make it difficult to rest or mentally prepare for the next step (29). The lack of calm areas or accessible retreat spaces particularly impacts some people with invisible disabilities or sensory sensitivities, for whom the physical environment itself becomes a source of stress or overload (26, 39). Even boarding areas open to all do not always offer armrest-equipped seating, flicker-free lighting, or tactile orientation aids on the floor, making it harder for some users to engage with the space.

Certain devices and elements within airport spaces, when poorly designed or ill-adapted, turn ordinary gestures into obstacles. A check-in counter that is too high, lacking a writing surface or an induction loop (an assistive listening system that transmits sound directly to hearing aids), becomes exclusionary; an automatic door that closes too quickly, a moving walkway without visual markers, or an escalator without an accessible alternative can make the journey stressful or unsafe (22, 31, 41). These elements send an implicit message to some people with disabilities: this place was not designed with you in mind (31). The appearance and visual organization of a space also influence how users perceive their presence and legitimacy within it. A clean, well-lit environment with clear and consistent signage communicates a sense of recognition and respect. In contrast, aging, improvised, or poorly maintained installations suggest a minimal, purely functional response, sometimes perceived as degrading (30). In many cases, accessibility features are technically present, but their placement and integration within the terminal layout reveal their marginal status, experienced as secondary additions relegated to the periphery of the usual travel path (26, 30). At the same time, some studies highlight efforts to improve the integration of accessibility features within mainstream passenger flows, reflecting a growing awareness of the importance of inclusive and dignified spatial design.

#### Affordability

In the included studies, for many persons with disabilities, every trip through an airport can entail additional expenses simply because the environment is not designed to allow them to move freely (23). From the moment of arrival, parking already raises concerns. The closest spots to terminal entrances, which are essential to reduce walking distances, are usually subject to higher fees. Some policies provide exemptions, but these are not always automatic and often require a specific process or supporting documentation that may be difficult to obtain on-site (26, 31). For a passenger who must carefully plan every movement, having to locate a kiosk, fill out a form, or wait for reimbursement represents an invisible yet very real barrier (23, 39).

Within the terminal, long distances between service zones sometimes require the use of internal transport solutions. If shuttles are unavailable, full, or inaccessible, the only alternatives may be to pay for a motorized wheelchair, hire a private service, or rely on a companion who may be paid to assist with navigating the terminal (23, 31). Access to basic services such as a place to sit, drink water, or rest in a quiet space is not always guaranteed free of charge. In many airports, comfortable seating is concentrated in private lounges, accessible rest areas are only available past security, and quiet zones are fee-based or integrated into premium services (30). A basic functional need such as sitting down, resting, or shielding oneself from noise becomes a privilege conditioned by purchasing power (26, 29).

When the physical environment is not readable or fluid, digital tools are supposed to compensate. However, check-in kiosks, assistance apps, or biometric devices are rarely free or truly universal. When they are not compatible with users’ personal assistive technologies or require reservations under strict conditions, sometimes up to 48hours in advance, they become restricted-access tools, pushing users toward costly or paid alternatives (25, 26). For some individuals, having to plan every step in advance under the risk of not receiving assistance turns what is meant to be a service into a financial constraint.

This cost does not affect only those with disabilities. Companions often have to pay to access restricted zones, obtain special passes, or park near the terminal. In some cases, they must purchase a partial ticket or pay entrance fees just to accompany a loved one in waiting areas (24, 37). The economic effort is thus compounded by a logistical and emotional investment rarely acknowledged or compensated.At the same time, some studies highlight efforts to improve financial accessibility, such as free assistance services or policy adjustments, although their implementation remains uneven across airport contexts.

#### Usability

In an airport, everything begins with a gesture: pressing a button, pushing a door, reading a sign, finding a seat. Actions that are simple and automatic for some. But for others, each interaction becomes a silent negotiation with an environment that seems to have been designed without them in mind (28, 41). Elevators may be present, but their control panels are too high to be reached without effort by a wheelchair user or a person of short stature. Automatic doors do open, but only if one can locate and activate a button hidden behind a decorative plant or placed in the middle of a blank wall (31, 38). At that moment, the promise of autonomy turns into waiting, dependence, or even resignation. So-called “accessible” restrooms are another example. Their signage is reassuring, but their interiors tell a different story: grab bars placed too far away, toilets too low, mirrors out of reach, faucets impossible to operate with trembling or painful hands (30, 39). At times, the issue is not a lack of facilities; rather, it lies in the failure to adapt these facilities to the real diversity of human bodies (5). A terminal flooded with harsh lighting, saturated with loudspeaker announcements, and filled with inconsistent pictograms can become a disorienting space for individuals with sensory or cognitive limitations (42, 43).

Even technology, despite its promise, can create distance. Automated check-in kiosks and biometric gates are becoming more widespread, often without considering the variety of user profiles. For some, these interfaces are a barrier: screens that move too quickly, complex navigation logic, and no voice or tactile alternatives (29, 30, 41). In the absence of inclusive design, the promise of independence turns into forced dependency (25, 35). Human assistance is then requested not by choice, but by necessity, revealing a breakdown in the interaction between user and system (24, 26, 30). Even assistive equipment, including wheelchairs, seats, and carts, falls short if it is not adapted to users’ needs. A waiting seat without armrests, too low, slippery, or placed in a high-traffic zone becomes unusable for an older adult or someone with joint pain (34, 37). A poorly maintained wheelchair, or one that is unavailable when needed, fails in its support function. At times, the key issue is not the presence of the equipment, but the actual possibility of using it (5).

## Discussion

This scoping review aimed to examine the physical environment of airports and how it shapes access for persons with disabilities, including its interaction with operational and service-related components. The results were presented in accordance with the five dimensions of access proposed by Fougeyrollas (5). However, in this section, the findings are discussed according to the different stages of the airport journey, while maintaining a link with the five dimensions of access.

### Arrival at the airport

In the included studies, accessibility appears to be compromised from the very first step: although ramps, drop-off zones, and reserved parking exist, they are often distant, poorly marked, or difficult to use without assistance (27, 34). This gap between regulatory compliance and actual usability forces travelers to navigate fragmented and stressful environments, undermining autonomy and dignity (9, 32). The situation is especially critical for those with invisible disabilities, who frequently feel the need to explain or justify themselves, impacting the acceptability of the experience (33, 34). Additionally, affordability emerges as a barrier when services such as accessible parking or priority assistance are subject to extra fees or reserved for premium passengers (22, 32). These findings suggest that arrival spaces remain shaped by a logic of flow rather than inclusion. Reimagining access at this stage requires environments that reduce cognitive and physical strain and affirm the presence of every traveler without requiring negotiation (27, 44). At the same time, some studies highlight ongoing efforts to improve the accessibility of arrival areas, including better signage and dedicated support services, although their implementation remains uneven across contexts.

### Entering the terminal and check-in

The terminal entrance, designed for openness, often presents subtle but persistent barriers that challenge both accessibility and usability. A heavy door, a high counter, or a poorly positioned handle can disrupt the experience for individuals using mobility aids or of short stature (9, 27, 44, 45). The check-in process amplifies these issues. While digital systems such as kiosks, apps, and biometrics are presented as improvements, they frequently exclude travelers unfamiliar with such tools, particularly older adults or those with cognitive impairments (22, 34). Usability is reduced, and the promised autonomy is replaced by dependence. The human dimension is equally inconsistent: interactions range from welcoming to dismissive, and for those with invisible disabilities, repeated explanations can create emotional fatigue and reduce the acceptability of the environment (9, 33). These findings reinforce the need for universal design principles that anticipate diverse user needs from the outset, ensuring smoother and more inclusive interactions across all access dimensions (44, 45). Ideally, check-in should facilitate departure and initiate a respectful journey where accessibility, usability, and dignity are explicit (9, 27). At the same time, several studies highlight improvements in digital services and assistance practices, suggesting a gradual evolution toward more inclusive check-in experiences.

### Security screening

In the included studies, the procedures involved scanners, physical inspections, and rapid verbal commands often presume normative physical and cognitive abilities (9, 27, 33, 34). Usability is compromised for individuals with mobility or sensory impairments who struggle to comply with these demands. These moments can also be psychologically taxing, especially when travelers must disclose private information without consent, affecting the acceptability of the experience (32, 33, 44). Technology, though intended to improve efficiency, can introduce further barriers when scanners demand unrealistic physical postures or raise privacy concerns (22, 34). Affordability also plays a role, longer delays or the need for personalized support may push travelers to purchase upgrades to reduce stress, turning access into an economic burden (32). Issues related to dignity are particularly at stake in these situations. Inclusive practices should not be limited to streamlining security procedures, but rather aim to reframe them as relational encounters by training personnel to recognize diverse bodies and minds and to ensure travelers are treated with empathy and respect (9, 27, 32). At the same time, some studies highlight ongoing efforts to improve accessibility in security procedures, including adapted screening protocols and increased staff awareness, although their implementation remains variable.

### Navigating and waiting in the terminal

In the included studies, findings suggest that accessibility appears fragmented lowered counters, tactile paths, or reserved seating areas often appear as isolated features rather than part of an integrated system (44, 45). This patchwork design undermines usability, especially when moving through the terminal requires ongoing adaptation or assistance (9, 27, 32). Signage, though technically compliant, may not be usable from a seated position or for those with visual or cognitive impairments when it is inconsistently placed or visually overloaded (34, 44, 45). For individuals with sensory or neurocognitive disabilities, constant noise, flashing lights, and crowded spaces compromise acceptability and create sensory overload (33, 34). Waiting areas, far from providing respite, often feature furniture that is too low, lacks armrests, and is situated in noisy environments, all of which reflect a design bias toward able-bodied users (9, 27, 44, 45). Furthermore, comfort and quiet are often commodified, accessible only through paid lounges or priority zones, introducing affordability challenges and reinforcing inequality (23, 32). A truly inclusive terminal must be designed to support rest, orientation, and independence without requiring travelers to justify their needs or pay for accessibility (9, 27, 32, 44). At the same time, several studies highlight improvements in wayfinding systems and the integration of accessibility features, suggesting a gradual shift toward more inclusive terminal environments.

### Boarding process

In the included studies, boarding is often described as the moment the journey truly begins. Yet for many travelers with disabilities, it is also when the experience of exclusion becomes most tangible (9, 27). Our findings suggest that travelers requiring assistance are often routed through side entrances or service areas that reinforce segregation rather than comfort (32, 44). In terms of usability and acceptability, these detours create delays, confusion, and emotional strain. The common principle of “first on, last off” is rarely respected, leading to frustration and a diminished sense of equity (9, 32). The transfer from a personal mobility device to an aircraft seat is particularly delicate, often conducted without adequate privacy or staff training, impacting both dignity and autonomy (9, 27). These observations highlight the relational nature of accessibility: infrastructure alone cannot ensure inclusion. What matters is how people are welcomed and supported. True accessibility means slowing down when necessary, adapting protocols, and ensuring each traveler is treated not as a logistical burden, but as a valued passenger deserving of care and respect (27, 32, 44). At the same time, some studies highlight improvements in boarding assistance and staff training, suggesting a gradual move toward more inclusive boarding practices.

### Arrival and baggage claim

In the included studies, although arrival should signal the end of the journey, it often becomes one of its most challenging moments, particularly in the baggage claim area (9, 44). Long walking distances, limited signage, and constant noise undermine usability, particularly for those with cognitive, sensory, or physical limitations (27, 45). Acceptability is compromised when assistive devices such as wheelchairs are mishandled, lost, or damaged. These items should not be regarded as ordinary luggage; they are essential extensions of the body that support autonomy and daily functioning. Their loss immediately induces dependence and emotional distress, violating the traveler’s dignity (9, 32). Fatigue also plays a crucial role. After navigating a journey full of obstacles, facing yet another disorienting environment can overwhelm even the most experienced traveler (9, 32). Yet this phase remains largely ignored in accessibility planning, despite being a vital part of the user journey. Our findings emphasize that accessibility must be conceived as a continuum: the ability to leave the airport with the same ease as entering it is a condition for a genuinely inclusive experience (27, 44). At the same time, some studies highlight improvements in baggage handling procedures and assistance services, suggesting growing attention to this final stage of the airport journey, although gaps remain.

### Cross-cutting themes and implications

Looking across the entire airport journey, our findings suggest that obstacles are not isolated issues, but rather symptoms of a system where access often continues to be treated as an adjustable variable, not as a structuring principle in the reviewed studies (9, 44). Each stage (i.e., airport arrival, check-in, security, navigation, boarding, and baggage claim) reveals similar tensions: infrastructures may be present, but they often fail to deliver on usability and autonomy. A recurring theme in the included studies is the dominance of compliance over actual usability. Whether it’s a ramp placed too far from the entrance, a kiosk too complex to navigate, or a scanner demanding impossible bodily positions, the mere presence of accessible features does not guarantee that travelers can use them effectively, independently, or with dignity (27, 32). This gap between design intent and user experience undermines both accessibility and acceptability at every step of the way.

Another transversal finding is the persistent overreliance on individual interventions rather than systemic solutions. From check-in to boarding, many travelers depend on the kindness of staff or strangers to overcome design limitations. While such gestures can be meaningful, they expose a precarious model of inclusion, where access is neither guaranteed nor consistent (9, 44). Usability in this context becomes contingent and unpredictable. The near-total absence of provisions for invisible disabilities reinforces these disparities. The included studies suggest that sensory hypersensitivities, neurocognitive limitations, and psychological distress are rarely accounted for in airport environments despite their profound impact on navigation, orientation, and emotional well-being (33, 34). This suggests that airport accessibility continues to be understood primarily through visible forms of disability, while the realities associated with invisible disabilities receive less attention in both infrastructure planning and service delivery. Similar trends have been observed in accessible tourism research, where mobility impairments have traditionally occupied a more prominent place than other disability experiences (6, 7).

This oversight undermines acceptability and usability, turning what should be neutral public spaces into stressful, exclusionary environments. Time emerges as a critical, often invisible, dimension of exclusion. Extended waiting, repeated explanations, and procedural delays affect nearly every phase of the journey, especially for those requiring assistance. This “expanded time” is not only a usability issue but also generates emotional fatigue and hidden financial costs, whether through missed connections or the need to pay for faster services (9, 32). Ultimately, our findings point to dignity as a foundational, yet particularly vulnerable, dimension of access. From the entrance to the baggage carousel, too many travelers report feeling scrutinized, exposed, or illegitimate, especially when they must repeatedly explain or justify their needs (27, 44). Inclusion will only be real when movement through the airport no longer requires constant negotiation of one’s legitimacy. These insights call for a deep rethinking of how accessibility is understood and practiced. Accessibility cannot remain the exclusive concern of specialized staff or be addressed only through isolated technical fixes. Instead, it must become a shared responsibility, embedded in architectural planning, service design, digital interfaces, and everyday interactions. Above all, access must be reframed not as a technical constraint, but as an ethical imperative grounded in the recognition of every traveler’s dignity and right to belong (9, 32).

These findings also point to broader questions of participation and social inclusion. The barriers encountered throughout the airport journey can affect not only how people move through airport environments, but also their ability to travel, access opportunities, and participate in everyday social, economic, and leisure activities. From this perspective, airport accessibility is not limited to the design of infrastructure and services. It is also linked to wider discussions on mobility rights, transport equity, and equitable participation in society for people with disabilities (46–48). These issues are particularly relevant in the context of tourism, where mobility is often a prerequisite for participation. Accessible tourism research has shown that barriers encountered throughout the travel process can influence not only access to destinations, but also the overall travel experience. Difficulties related to navigation, information, assistance services, and airport procedures identified in this review may therefore limit participation in tourism activities for some travelers with disabilities. As key points of entry and departure, airports play an important role in shaping these experiences (6, 7). At the same time, several studies highlight promising practices and ongoing improvements, including the integration of digital wayfinding tools, the development of dedicated assistance services, and increasing attention to universal design principles. These initiatives suggest a gradual shift toward more inclusive airport environments, although their implementation remains uneven across contexts.

### Limitations

Every scientific endeavor, however rigorous, carries its own limitations. Acknowledging these is not a sign of weakness; on the contrary, it reflects intellectual honesty and respect for the complexity of reality (43, 49). The first limitation of this review lies in its geographical and cultural scope. A significant portion of the included studies originates from Europe, North America, or East Asia. Yet, accessibility is deeply intertwined with socio-cultural, economic, and regulatory contexts. What might be deemed acceptable or even desirable in a European airport may be experienced very differently in an African, South American, or Middle Eastern airport. This reality is well documented in research on the diversity of accessibility policies, which emphasizes the substantial variability of practices across geopolitical contexts (32, 50). It is therefore likely that certain specific challenges or, conversely, some ingenious local solutions are still missing from this overview (32). A related limitation concerns the gray literature included in this review. While the scientific studies were drawn from a range of international contexts, the gray literature was limited to Canadian sources. This choice helped document current accessibility policies, regulations, and practices within the Canadian airport sector. However, these documents reflect a specific regulatory and institutional context that may differ from those of other countries. Consequently, some observations drawn from the gray literature may not be fully transferable to other airport systems.

Another limitation relates to the very lens through which research views disability. The included studies focus predominantly on visible physical disabilities. Realities connected to invisible disabilities, cognitive limitations, or psychological conditions remain relatively under-documented in the literature, even though they profoundly shape the lived experience in complex environments such as airports (27, 33). This observation is also frequent in critical analyses of disability literature, which point to an overrepresentation of physical disabilities at the expense of other forms of impairment (51). This absence creates a blind spot that risks perpetuating a model of access designed for a few typical profiles, neglecting the plurality of lived experiences (33). It is also important to recognize the limitations inherent to the nature of the included studies. Many are qualitative, rich in human detail, but sometimes limited in statistical representativeness (49, 52). Other studies adopt highly technical approaches but overlook the subjective experiences of travelers. Finally, this review is constrained by its temporality. It captures a snapshot of the situation at a given moment. Yet, access is a constantly evolving field, shaped by legislation, technological innovations, and, above all, civic activism (32, 50). What is observed as a shortcoming today might already be in the process of transformation tomorrow, and conversely, recent advances can prove fragile or short-lived if not consolidated (52).

## Conclusion

Airport access cannot be reduced to regulatory compliance or the mere presence of physical infrastructures like ramps and elevators. This scoping review suggests that, despite growing awareness and notable progress, the air travel experience for people with disabilities remains marked by persistent barriers. Access often continues to rely on assistance-based models in the reviewed literature, emphasizing compensation over autonomy and reflecting a broader system where disability is managed rather than anticipated through inclusive design. This reliance results in fragmented, inconsistent experiences across airports, shaped by divergent organizational practices, economic constraints, and a lack of unified international standards (9, 44).

By applying the conceptual framework of the five dimensions of access, availability, accessibility, acceptability, affordability, and usability (5) this review highlights a recurring gap between theoretical intentions and actual implementation. Infrastructures may be in place, but their integration into real-world passenger flows, their adaptability to varied functional profiles, and their usability in practice remain limited and uneven. Universal design principles, though increasingly referenced, are still rarely embedded across the full spectrum of airport architecture, technologies, and procedures (26, 44).

Moreover, the findings underscore that access is not a fixed attribute but a relational and evolving experience shaped by interactions between individuals and the built environment. Accessibility must be understood as a continuum extending from arrival at the airport to the final moment of departure, and as a principle embedded in every stage of the journey. Rethinking access involves more than retrofitting. It requires a shift in culture, where inclusion becomes a guiding logic from the outset. Technological innovations may support this transition, but only if designed inclusively and deployed in ways that preserve autonomy and respect privacy (9, 36).

At the same time, the reviewed literature highlights emerging initiatives and promising practices aimed at improving accessibility, suggesting a gradual shift toward more inclusive airport environments, although important gaps remain. Future research is essential to assess the lived impact of current measures, identify context-specific obstacles, and co-design responses with those most affected. Ensuring that airports become truly inclusive spaces, welcoming everybody and every mind, is not only a matter of functionality but a fundamental expression of human dignity and social justice (5, 44).

## Data Availability

The datasets presented in this study can be found in online repositories. The names of the repository/repositories and accession number(s) can be found in the article/Supplementary Material.

## Appendix 1

**Table A1:** Characteristics and key findings of included studies.

#	Study & location	Aim	Methodology & population	Airport context	Key findings
1	Acar and Nur (53) – Türkiye	To assess airport service accessibility for passengers with disabilities.	Quantitative survey (*n*=155).	Ankara Esenboğa Airport.	Accessibility services were generally rated positively, with differences across demographic groups
2	Arcidiacono et al. (24) – Italy	Analyze PRM assistance system using Axiomatic Design	Case study; participants: PRM system analysis	Major Italian tourist airport	Assistance fragmented and design-dependent; framework identifies inefficiencies
3	Atkin et al. (26) – United Kingdom	Explore mobility-related barriers across travel journey	Qualitative study (4 focus groups, *n*=15); participants: people with visible and invisible disabilities	UK airports	Issues include unsafe transfers, lack of toilets, inconsistent staff training
4	Cerdan Chiscano (42) – Spain	Co-design inclusive airport experience for autistic children	Qualitative study (*n*=25 families); participants: children with autism and families	Barcelona–El Prat Airport	Sensory overload and unpredictability are key barriers; co-design improves experience
5	Chang and Chen (22) – Taiwan	Evaluate service quality gaps using IPA model	Survey study (*n*=130); participants: individuals with lower extremity impairments	Airports in Taiwan	Gaps between importance and satisfaction; need for better physical infrastructure
6	Chang and Chen (23) – Taiwan	Assess staff perceptions of disability services	Quantitative survey (*n*=180 staff); participants: airport, airline, and government staff	Taiwan Taoyuan International Airport	Lack of training and accessible facilities; gender differences in willingness to assist
7	Darvishy et al. (35) – Switzerland	Develop navigation system for visually impaired travelers	Technical development study; participants: simulation-based	Simulated airport environments	Mobile navigation concept enhances autonomy; scalable to transport systems
8	Guerreiro et al. (34) – USA	Evaluate BLE-based navigation system for visually impaired users	Mixed study (focus groups *n*=9 + field study *n*=10); participants: visually impaired users	Pittsburgh International Airport	Users navigated independently; need for real-time updates and POI access
9	Liu et al. (36) – USA	Co-design mobile app for airport navigation	User-centered design study (*n*=12); participants: older adults and persons with disabilities	Austin–Bergstrom International Airport	Real-time information critical; supports autonomy; universal design essential
10	Oostveen and Lehtonen (41) – European Union	Assess accessibility of automated border control systems	Mixed-methods study (survey *n*=139 + interviews *n*=14); participants: persons with disabilities and stakeholders	European airports (ABC systems)	ABC gates largely inaccessible; accessibility seen as a rights issue
11	Peterson et al. (43) – USA	Examine travel challenges for persons with dementia	Mixed-methods study (*n*=224); participants: persons with dementia + companions	U.S. airports (general)	Major issues include anxiety, navigation confusion, lack of quiet spaces
12	Qing et al. (40) – USA	Evaluate mobile wayfinding tool using simulation	Experimental study (*n*=24); participants: wheelchair users	St. Louis Lambert International Airport (simulated)	App reduced anxiety but had limited impact on travel time
13	Tata et al. (33) – Australia/UK	Analyze experiences of hidden disabilities via online reviews	Netnographic study (*n*=203 reviews); participants: passengers with invisible disabilities	Mainly UK airports	Key issues: poor staff interaction, lack of support, noisy environments
14	Yücel and Polat (28) – Türkiye	Evaluate accessibility of barrier-free airport	Qualitative case study (*n*=10); participants: passengers with disabilities	Erzincan Airport	Good compliance but inconsistent tactile paths and signage
